# Fate of dissected arch vessels by adventitial inversion technique for acute type A aortic dissection repair

**DOI:** 10.1093/icvts/ivac185

**Published:** 2022-06-27

**Authors:** Yuriko Takeuchi, Ryo Suzuki, Hiroshi Kurazumi, Ryosuke Nawata, Toshiki Yokoyama, Sarii Tsubone, Yutaro Matsuno, Akihito Mikamo, Kimikazu Hamano

**Affiliations:** Department of Surgery and Clinical Science, Yamaguchi University Graduate School of Medicine, Ube, Yamaguchi, Japan; Department of Surgery and Clinical Science, Yamaguchi University Graduate School of Medicine, Ube, Yamaguchi, Japan; Department of Surgery and Clinical Science, Yamaguchi University Graduate School of Medicine, Ube, Yamaguchi, Japan; Department of Surgery and Clinical Science, Yamaguchi University Graduate School of Medicine, Ube, Yamaguchi, Japan; Department of Surgery and Clinical Science, Yamaguchi University Graduate School of Medicine, Ube, Yamaguchi, Japan; Department of Surgery and Clinical Science, Yamaguchi University Graduate School of Medicine, Ube, Yamaguchi, Japan; Department of Surgery and Clinical Science, Yamaguchi University Graduate School of Medicine, Ube, Yamaguchi, Japan; Department of Surgery and Clinical Science, Yamaguchi University Graduate School of Medicine, Ube, Yamaguchi, Japan; Department of Surgery and Clinical Science, Yamaguchi University Graduate School of Medicine, Ube, Yamaguchi, Japan

**Keywords:** Aortic dissection, Aortic arch replacement, Arch vessel, Adventitial inversion technique

## Abstract

**OBJECTIVES:**

The adventitial inversion technique is used widely for aortic reconstruction for acute type A aortic dissection, as it easily controls the bleeding at anastomotic sites and closes the patent false lumen. However, this technique for arch vessel reconstruction has not been previously reported. Therefore, we applied the adventitial inversion technique for dissected arch vessel reconstruction to close the patent false lumen.

**METHODS:**

Among 57 consecutive patients who underwent emergency surgical treatment for acute type A aortic dissection from July 2006 to July 2012, the adventitial inversion technique for the dissected arch vessels was performed in 26 patients (42 arch vessel stumps). The patency and morphologic change of the false lumen of the arch vessels were evaluated using contrast-enhanced computed tomography.

**RESULTS:**

Overall, 2 hospital deaths were recorded, and the hospital mortality rate was 4%. No postoperative cerebral strokes and reoperations due to bleeding occurred. Follow-up by contrast-enhanced computed tomography was completed in 24 patients (37 stumps) with a mean duration of 99 ± 35 months. The postoperative closure rate of the false lumen after adventitial inversion was 86%, which was higher than when adventitial inversion was not used. No adverse events including stroke occurred during follow-up period.

**CONCLUSIONS:**

This technique facilitates the closure of the false lumen of dissected arch vessels and might improve clinical outcomes.

**Figure:**
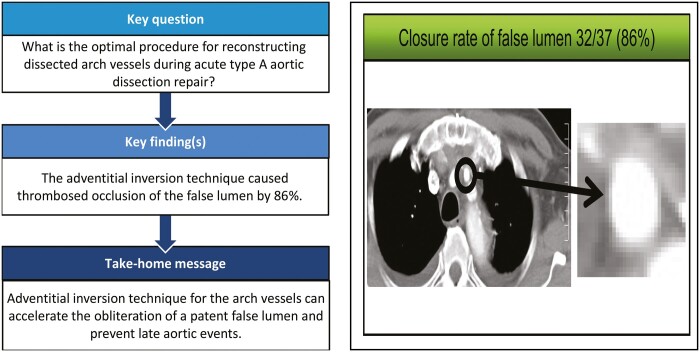

## INTRODUCTION

For the surgical treatment of acute type A aortic dissection (AAAD), preventing anastomotic complications, such as bleeding and dehiscence due to friable tissues at the anastomotic sites, is essential. Moreover, facilitating closure or obliterating the false lumen of the dissected aortic wall is necessary because persistent patent false lumen is considered an important factor that compromises the long-term prognosis after surgical repair of AAAD [1–5].

The adventitial inversion technique (AIT) was first reported in 1995 by Floten *et al.* [6] to reinforce the remainder of the aorta at the anastomotic site and reduce anastomotic complications such as bleeding and dehiscence. The AIT has been reported to obliterate the false lumen of the aorta and to excellently control bleeding. Folding the adventitia inward ensures that the false lumen is excluded from antegrade flow. This idea has been supported by several studies demonstrating the superiority of this technique for the dissected aortic wall to some other methods, such as the sandwich technique [7, 8]. Furthermore, this technique has reduced the patency of the dissected aortic false lumen in the long-term postoperative period [9].

The AAAD is frequently complicated with the dissection of arch vessels. Even after a total arch replacement was performed with the reconstruction of the arch vessels, a brachiocephalic branch re-entry occurred in 40% of cases, and false lumens were patent postoperatively with a relatively high percentage [10]. Residual arch vessel dissection may cause ischaemic brain events. Therefore, eliminating the false lumen would be ideal during surgery for AAAD. However, the usage of AIT for reconstructing dissected arch vessels and the fate of the false lumen of arch vessels reconstructed through this procedure have not been reported. We have performed the AIT to reconstruct dissected arch vessels, as well as proximal and distal aortic stumps since July 2006. Hence, this study aimed to present our 10-year clinical results of patients who were received the AIT for their dissected arch vessels including cerebral events, focusing on the morphological change of the false lumen of the arch vessels evaluated by contrast-enhanced computed tomography (CT) scans.

## PATIENTS AND METHODS

### Ethical statement

This retrospective study was approved by the institutional review board of Yamaguchi University Hospital (2020-149), and the study was conducted in accordance with the Declaration of Helsinki.

### Patients

**Figure 1:**
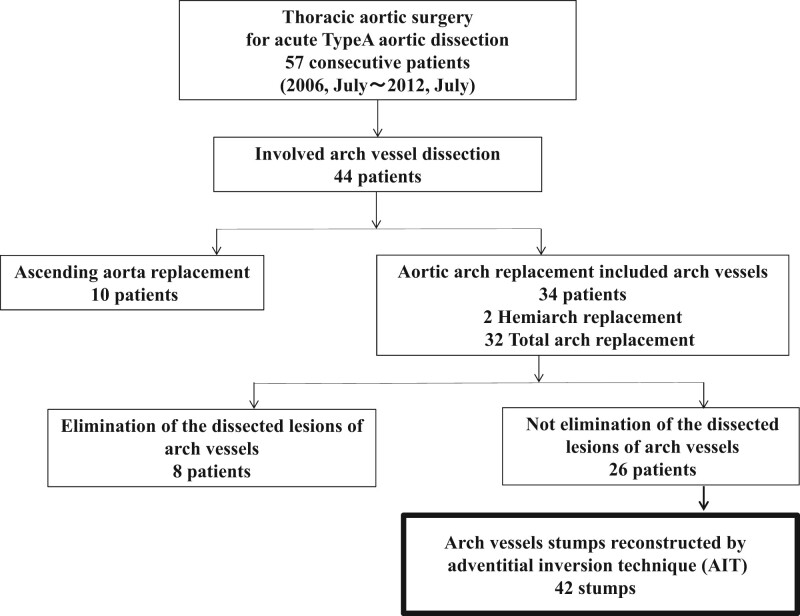
Details of the patients who had arch vessel dissection.

Altogether, 57 consecutive patients who underwent emergency surgical treatment for AAAD from July 2006 to July 2012 were included in this study. Figure 1 depicts the preoperative status of the arch vessels in the 57 patients. Of the 57 patients, 44 patients had dissected arch vessels, and 34 of the 44 underwent aortic arch replacement including arch vessels (hemiarch replacement: 2/34, total arch replacement: 32/34), while residual 10 patients underwent ascending aortic replacement. Of the 34 patients, the dissected lesions of the arch vessels were closed in 8 patients, while in 26 patients, their arch vessels were dissected at the far distal side. Therefore, 42 dissected arch vessel stumps of 26 patients were reconstructed at the dissected site using the AIT. We investigated these 46 stumps of the 26 patients, focusing on the morphological change of the false lumen.

### Surgical techniques

During the study period, we did not change the basic process and the surgical technique. In all the patients, intraoperative transoesophageal echocardiography was monitored. The surgery began with a median sternotomy, followed by exposure of the bilateral axillary arteries. Extracorporeal circulation was established with arterial cannula placed in the both axillary arteries, bicaval cannulation in the superior and inferior vena cava, and a left ventricular venting cannula placed via the right superior pulmonary vein. Systemic cooling was initiated, with a target rectal temperature of 25°C. After clamping the arch vessels, selective cerebral perfusion was initiated with bilateral axillary arteries. The ascending aorta was transected, and direct antegrade cardioplegia was infused. Selective cerebral perfusion by 3 vessels was started after by adding the cannulation of the left common carotid artery. The primary intimal tear was resected if it was present in the ascending aorta, aortic arch and proximal descending thoracic aorta. Replacement with graft was launched from the distal aortic stump using AIT. The adventitia was completely separated from the media, and the adventitia was trimmed 10–15 mm proximal to the intimal end. The short straight graft (5–7 cm) was inserted to the descending aorta as a neo-intima. The adventitia was inverted inside over the intima and tacked with 5-0 polypropylene interrupted mattress sutures. Distal anastomosis was performed proximal to the tacked line with a 4-0 polypropylene suture. After completing the distal anastomosis, the lower body circulation was restarted through the side branch of the graft. During the rewarming period, reconstruction was followed to the left subclavian artery, left cerebral artery and brachiocephalic artery, sequentially. If the arch vessels were dissected, we attempted to eliminate the dissected parts. However, when the dissection of the arch vessels extended to the far distal side and could not be eliminated, an AIT was also applied to the arch vessels. The trimmed adventitia was inverted into the true lumen and tacked with 5-0 polypropylene interrupted mattress sutures to fix the aortic wall without using internal Teflon felt or biological glue. To prevent lumen narrowing, careful detachment of the fatty tissue adhering to the adventitia results in only a very thin layer, and fixation sites are kept to the necessary minimum, with a maximum of 4 stitches even when the entire circumference is fixed. The anastomosis between the arch vessels and grafts was performed proximal to the tacked line with a 5-0 polypropylene suture. Subsequently, the selective cerebral perfusion was discontinued. With the exception of some patients who required aortic valve spring root replacement or Bentall operation, proximal reinforcement of the false lumen was performed by AIT in the same manner described in the distal aortic stump. The proximal anastomosis was performed distally to the tacked line. Weaning from extracorporeal circulation was done by infusing the terminal warm blood cardioplegia through the cardioplegic needle punctured to the graft.

### Follow-up

Preoperative contrast-enhanced CT scans and operation records of 26 patients were retrospectively analysed to reveal the preoperative status of arch vessels which were reconstructed using the AIT. The postoperative changes of the false lumen of arch vessels were evaluated by comparing postoperative contrast-enhanced CT scans performed before discharge and distant period after operation.

### Evaluation of the status of the false lumen by contrast-enhanced computed tomography scans

**Figure 2:**
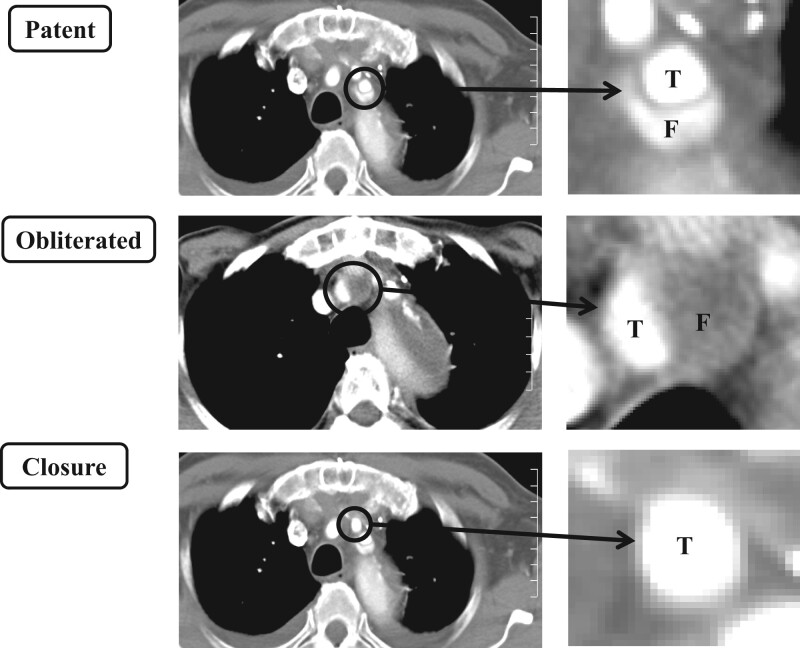
Three types of false lumen evaluated by computed tomography. Representative computed tomography images of patent, obliterated and closed false lumens.

We traced the blood flow in the false lumen of the arch vessels by observing its presence on CT. The false lumen was considered ‘patent’ if it was completely enhanced on CT, ‘obliterated’ if it existed but was not enhanced until the delayed phase due to thrombus formation in the false lumen and ‘closed’ when the false lumen disappeared, with the true lumen remaining. Figure 2 illustrates the abovementioned statuses of the arch vessels by exhibiting the actual contrast-enhanced CT scans.

### Statistical analysis

Continuous data are expressed as a mean ± standard deviation, and categorical data are expressed as a number and percentage. Survival data and freedom from cerebral events were analysed with standard Kaplan–Meier techniques for estimation of freedom from survival or cerebral stroke probabilities, and the time origin was time of surgery. All computations were performed using JMP Pro 15.0.0 (SAS Institute Inc., Cary, NC, USA).

## RESULTS

### Preoperative characteristics of the patients

Among the 57 patients who underwent thoracic aortic surgery for AAAD between July 2006 and July 2012 in our hospital, 26 patients (61%) underwent arch vessel reconstruction with the application of AIT. Table 1 lists the preoperative characteristics of these patients. Among them, 2 patients had clinical features of the Marfan syndrome. The duration between dissection onset and the beginning of surgery was 8.5 ± 4.5 h.

**Table 1:** Preoperative characteristics

	(*N* = 26)
Age (years)	62 ± 12
Male:female (*n*)	12:14
Weight (kg)	60.2 ± 16.4
Hb (g/dl)	12.6 ± 1.7
eGFR (ml/min/1.73 m^2^)	72.9 ± 30.2
Hypertension (*n*, %)	19 (73%)
Dyslipidaemia (*n*, %)	3 (12%)
Diabetes mellitus (*n*, %)	1 (4%)
History of smoking (*n*, %)	12 (46%)
Malperfusion	
Coronary	1 (4%)
Cerebral	2 (8%)
Renal	2 (8%)
Lower extremity	1 (4%)
Shock	0 (0%)
Aortic insufficiency	2 (8%)
Cardiac tamponade	0 (0%)

eGFR: estimated glomerular filtration rate; Hb: haemoglobin.

### Perioperative data

The mean extracorporeal circulation time was 358 ± 71 min, the mean aortic clamp time was 280 ± 63 min, the mean selective cerebral perfusion time was 190 ± 34 min and the mean cardiac arrest time was 117 ± 24 min. The primary entry was successfully excised in all of the 26 patients.

### Surgical mortality and postoperative complications

Two hospital deaths were recorded due to low output syndrome: one patient had acute myocardial infarction and the other had rupture of dissected aorta at onset. No postprocedural cerebral or brachial malperfusions and reoperations due to bleeding occurred.

### Follow-up by contrast-enhanced computed tomography scans

**Figure 3:**
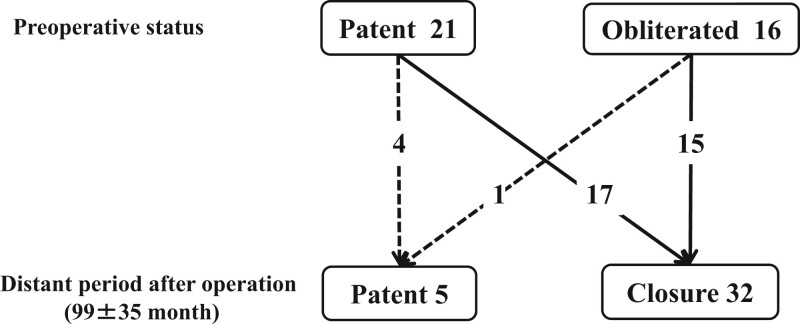
The changes in the arch vessel of the false lumens. There were 21 patent and 16 obliterated false lumens preoperatively. Of the 21 patent false lumens, 17 (81%) were closed whereas 4 (19%) remained patent. Of the 16 obliterated false lumens, 15 (94%) were closed whereas 1 (6%) converted to patent in the immediate postoperative period and remained patent in the long-term postoperative period.

According to preoperative contrast-enhanced CT scans, in 42 dissected arch vessels which would be reconstructed using the AIT, 24 were brachiocephalic arteries, 13 were left carotid arteries and 5 were left subclavian arteries. Contrast-enhanced CT scans were performed immediately postoperatively and few years after operation, except for in the 2 cases of mortality. Thus, we evaluated 37 stumps from 24 patients using contrast-enhanced CT. The mean CT follow-up period was 99 ± 35 months (23–158 months). The preoperative contrast-enhanced CT scans of 24 patients revealed 21 patent and 16 obliterated false lumen stumps. Of the 21 patent false lumen stumps, 17 (81%) stumps became closed whereas 4 (19%) stumps remained patent. Additionally, of the 16 obliterated false lumen stumps, 15 (94%) stumps remained closed whereas 1 (6%) stump became patent immediately postoperatively and remained patent in the long-term postoperative period (Fig. 3). Therefore, the closure rate of the false lumen in the long-term postoperative period was 86% (32/37).

Overall, 5 vessels had patent false lumen stumps in the long-term postoperative period. All 5 vessels had tears to their false lumen from the true lumen at the anastomotic sites. Moreover, of the 5 false lumen stumps, 3 false lumen stumps did not indicate any changes in width and length of the false lumen from the preoperative period to long-term postoperative period. Meanwhile, 2 false lumen stumps were enlarged chronologically (postoperative diameter/preoperative diameter = 1.39). However, no further surgical treatment was needed for the enlarged false lumen because the size was not sufficiently large for treatment.

### Follow-up by cerebral events and overall survival rates

**Figure 4:**
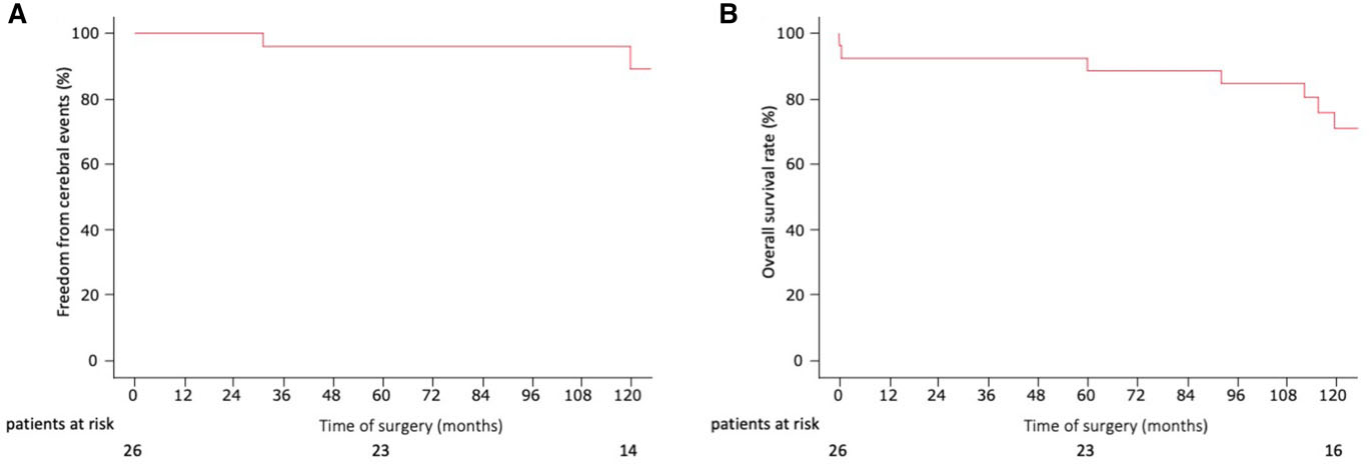
(**A**) Cerebral event-free rate of the patients. (**B**) The postoperative survival rates.

The cerebral event-free rates of the patients are shown in Fig. 4A and the postoperative survival rates are shown in Fig. 4B. In the patients who underwent total aortic arch replacement, the 5- and 10-year cerebral event-free rates were 96% and 89%, respectively, and the 5- and 10-year survival rates were 89% and 71%, respectively.

## DISCUSSION

The superiority of AIT for obliterating the aortic false lumen has been frequently reported [6–8]. However, applying AIT for dissected arch vessels in particular has not been previously reported. There are mainly 2 merits of this technique: easy haemostasis at the anastomotic sites and reduction of the patency rates of the false lumen. Concerning the adverse events due to the presence of a patent false lumen, Zieliński *et al.* have reported that the patent false lumen might not to be a source of stroke [11, 12], while other studies have reported otherwise [13–16]. However, the relationship between the patent false lumen and brain ischaemia remains unclear. Here, we revealed the patent false lumen rate of the cervical branches after total aortic arch replacement, and the 10-year survival and cerebral event-free rates of the patients. To the best of our knowledge, this is the first study to suggest a relationship between false lumen patency of the cervical branches and cerebral events. In our study, no relationship was identified between these factors. However, the patency of the false lumen should be reduced as much as possible because the patent false lumen of the dissected arteries may increase the risk of ischaemic events [13]. Here, the closure rate of the false lumen of the arch vessels was 86%, and the 10-year cerebral event-free rate was 89%. Although no significant difference was observed, a high false lumen closure rate may have reduced cerebral events. Furthermore, the excellent haemostasis at the anastomotic sites with the AIT is not doubtful. Therefore, applying the AIT would be beneficial despite a longer operative time.

Moreover, the AIT for the dissected arch vessels can be performed safely with no adverse events such as postprocedural cerebral and brachial malperfusion. In this study, there were 2 patients with neurological dysfunction due to dissection of the carotid artery or brachiocephalic arteries at onset, and the procedure had a closure rate of 86% for patent or obliterated false lumens at a distant point. The exact closure rate of the dissected arch vessels with the patent false lumen without AIT has not been previously reported, although the appropriate closure rates of 14–32% were calculated from some papers [12, 13]. Therefore, the closure rate using AIT can be considered very high at 86%.

Regarding the postoperative patent false lumens of the arch vessels in this study, we deemed that the unchanged patent false lumen through the observation period had 2 or more tears, and the enlarged one had only one tear, at the postoperative period. Two arch vessels that were patent postoperatively were enlarged, although the enlarged one had not reached the level that requires treatment. The aortic anastomotic technique or obliterating the false lumen of the distal aorta was discussed; however, the arch vessel reconstruction technique has been not fully considered. The AIT intends to protect the fragile intima by adventitia and prevent bleeding by covering the inside and outside with a strong adventitia. Preventing false lumen patency and aneurysms can be possible by creating a new entry, although careful follow-up is necessary. Nevertheless, these same events may occur during surgery even without applying the AIT.

More importantly but unexpectedly, patent false lumens reconstructed using the AIT were associated with new tears at the anastomotic site in all postoperative patent cases. The AIT may reinforce the vessel wall, although in some cases, a new entry might be created due to this procedure. Therefore, these are further issues that should be resolved in future studies.

## CONCLUSIONS

The AIT for reconstructing dissected arch vessels can be performed safely with no postoperative complications such as postprocedural cerebral ischaemia. Although the technique might cause enlargement of the false lumen and occurrence of a new tear at the anastomotic site in some cases, it may be an effective method to facilitate the closure of the false lumen in many cases.

**Conflict of interest:** none declared.

## Data availability statement

The data that support the findings of this study are available from the corresponding author upon reasonable request.

## Author contributions

**Yuriko Takeuchi:** Data curation; Formal analysis; Software; Visualization; Writing—original draft. **Ryo Suzuki:** Conceptualization; Data curation; Formal analysis; Investigation; Project administration; Resources; Supervision; Validation; Visualization; Writing—review & editing. **Hiroshi Kurazumi:** Conceptualization; Data curation; Formal analysis; Investigation; Resources; Visualization. **Ryosuke Nawata:** Data curation; Software; Visualization. **Toshiki Yokoyama:** Data curation; Software; Visualization. **Sarii Tsubone:** Data curation; Software; Visualization. **Yutaro Matsuno:** Data curation; Software; Visualization. **Akihito Mikamo:** Conceptualization; Data curation; Investigation; Methodology; Project administration; Resources; Supervision; Validation; Writing—review & editing. **Kimikazu Hamano:** Conceptualization; Funding acquisition; Investigation; Project administration; Resources; Supervision; Validation; Writing—review & editing.

## Reviewer information

Interactive CardioVascular and Thoracic Surgery thanks Michael Grimm, Gabriel Weiss and the other anonymous reviewer(s) for their contribution to the peer review process of this article.

## ABBREVIATIONS

AIT: Adventitial inversion technique
AAAD: Acute type A aortic dissection
CT: Computed tomography

## References

[1] Immer FF , Hagen U, Berdat PA, Eckstein FS, Carrel TP. Risk factors for secondary dilatation of the aorta after acute type A aortic dissection. Eur J Cardiothorac Surg 2005;27:654–71. 15784368 10.1016/j.ejcts.2004.11.031

[2] Moore NR , Parry AJ, Trottman-Dickenson B, Pillai R, Westaby S. Fate of the native aorta after repair of acute type A dissection: a magnetic resonance imaging study. Heart 1996;75:62–6. 8624875 10.1136/hrt.75.1.62 PMC484224

[3] Fattori R , Bacchi-Reggiani L, Bertaccini P, Napoli G, Fusco F, Longo M et al Evolution of aortic dissection after surgical repair. Am J Cardiol 2000;86:868–72. 11024403 10.1016/s0002-9149(00)01108-5

[4] Bernard Y , Zimmermann H, Chocron S, Litzler JF, Kastler B, Etievent JP et al False lumen patency as a predictor of late outcome in aortic dissection. Am J Cardiol 2001;87:1378–82. 11397357 10.1016/s0002-9149(01)01556-9

[5] Driever R , Botsios S, Schmitz E, Donovan J, Vetter HO. Long-term effectiveness of operative procedures for Stanford type A aortic dissections. Cardiovasc Surg 2003;11:265–72. 12802261 10.1177/096721090301100404

[6] Floten HS , Ravichandran PS, Furnary AP, Gately HL, Starr A. Adventitial inversion technique in repair of aortic dissection. Ann Thorac Surg 1995;59:771–2. 7887736 10.1016/0003-4975(94)01018-8

[7] Kim SW , Sung K, Lee YT, Kim WS, Park PW, Jun T-G et al Aortic false lumen patency following the adventitial inversion technique for acute DeBakey type I aortic dissection. J Card Surg 2010;25:548–53. 20678107 10.1111/j.1540-8191.2010.01099.x

[8] Tanaka K , Morioka K, Li W, Yamada N, Takamori A, Handa M et al Adventitial inversion technique without the aid of biologic glue or Teflon buttress for acute type A aortic dissection. Eur J Cardiothorac Surg 2005;28:864–9. 16275115 10.1016/j.ejcts.2005.08.029

[9] Oda T , Minatoya K, Sasaki H, Tanaka H, Seike Y, Itonaga T et al Adventitial inversion technique for type A aortic dissection distal anastomosis. J Thorac Cardiovasc Surg 2016;151:1340–5. 26856471 10.1016/j.jtcvs.2016.01.018

[10] Yasuda S , Imoto K, Uchida K, Karube N, Minami T, Goda M et al Evaluation and influence of brachiocephalic branch re-entry in patients with type A acute aortic dissection. Circ J 2017;81:30–5. 10.1253/circj.CJ-16-0462 27885195

[11] Zieliński T , Wołkanin-Bartnik J, Janaszek-Sitkowska H, Biederman A, Rynkun D, Makowiecka-Cieśla M et al Persistent dissection of carotid artery in patients operated on for type A acute aortic dissection—carotid ultrasound follow-up. Int J Cardiol 1999;70:133–9. 10454301 10.1016/s0167-5273(99)00072-8

[12] Charlton-Ouw KM , Azizzadeh A, Sandhu HK, Sawal A, Leake SS, Miller CC et al Management of common carotid artery dissection due to extension from acute type A (DeBakey I) aortic dissection. J Vasc Surg 2013;58:910–6. 23664277 10.1016/j.jvs.2013.03.042

[13] Neri E , Sani G, Massetti M, Frati G, Buklas D, Tassi R et al Residual dissection of the brachiocephalic arteries: significance, management, and long-term outcome. J Thorac Cardiovasc Surg 2004;128:303–12. 15282469 10.1016/j.jtcvs.2004.02.030

[14] Nagamine H , Ueno Y, Ueda H, Saito D, Tanaka N, Miyazaki M et al A new classification system for branch artery perfusion patterns in acute aortic dissection for examining the effects of central aortic repair. Eur J Cardiothorac Surg 2013;44:146–53. 23242985 10.1093/ejcts/ezs631

[15] Tanaka H , Okada K, Yamashita T, Morimoto Y, Kawanishi Y, Okita Y. Surgical results of acute aortic dissection complicated with cerebral malperfusion. Ann Thorac Surg 2005;80:72–6. 15975343 10.1016/j.athoracsur.2004.12.049

[16] Estrera AL , Garami Z, Miller CC, Sheinbaum R, Huynh TT, Porat EE et al Cerebral monitoring with transcranial Doppler ultrasonography improves neurologic outcome during repairs of acute type A aortic dissection. J Thorac Cardiovasc Surg 2005;129:277–85. 15678036 10.1016/j.jtcvs.2004.08.052

